# Evaluation of Phytochemical Constituents, Antioxidant Potential, and Toxicological Profile of Selected Medicinal Plants from Romania’s Spontaneous Flora

**DOI:** 10.3390/molecules31091527

**Published:** 2026-05-04

**Authors:** Lidia-Ioana Virchea, Cecilia Georgescu, Adina Frum, Endre Máthé, Monica Mironescu, Bence Pecsenye, Robert Nagy, Oana Viorica Danci, Maria-Lucia Mureșan, Maria Totan, Felicia-Gabriela Gligor

**Affiliations:** 1 Faculty of Medicine, “Lucian Blaga” University of Sibiu, Lucian Blaga Str. 2A, 550169 Sibiu, Romania; lidia.virchea@ulbsibiu.ro (L.-I.V.); maria.muresan@ulbsibiu.ro (M.-L.M.); maria.totan@ulbsibiu.ro (M.T.); felicia.gligor@ulbsibiu.ro (F.-G.G.); 2 Faculty of Agriculture Sciences, Food Industry and Environmental Protection, “Lucian Blaga” University of Sibiu, Dr. Ion Rațiu Str. 7-9, 550012 Sibiu, Romania; monica.mironescu@ulbsibiu.ro; 3 Institute of Nutrition Science, Faculty of Agricultural and Food Sciences and Environmental Management, University of Debrecen, Böszörményi Str. 128, 4032 Debrecen, Hungary; endre.mathe@agr.unideb.hu (E.M.); pecsenye.bence@agr.unideb.hu (B.P.); nagy.robert@agr.unideb.hu (R.N.); 4 Department of Life Sciences, Faculty of Medicine, Vasile Goldis Western University of Arad, L. Rebreanu Str. 86, 310414 Arad, Romania; 5 Faculty of Sciences, “Lucian Blaga” University of Sibiu, Dr. Ion Rațiu Str. 5-7, 550012 Sibiu, Romania; oana.danci@ulbsibiu.ro

**Keywords:** HPLC, phenolic compounds, antioxidant, *Drosophila melanogaster* toxicity, *Achillea millefolium*, *Mentha longifolia*, *Thymus serpyllum*, yarrow, wild mint, wild thyme

## Abstract

The aim of this study was to analyze the composition and dual beneficial and toxic effects of *Achillea millefolium* L., *Mentha longifolia* L., and *Thymus serpyllum* L. extracts. The phenolic profile, total phenolic content (TPC), antioxidant activity, and drosopterin eye content (DEC) were determined by modern methods. The viability and developmental time of *D. melanogaster* were assessed by a diet-dependent viability test. The results show that the phenolic profile varied depending on the extract type and plant species. The TPC ranged between 5.32 and 29.32 mg GAE/g dry weight. All the plant extracts exert antioxidant effect in the applied in vitro tests. In the case of *D. melanogaster* fed with a normal diet supplement with different concentrations of the plant *A. millefolium* L. extract, a biphasic effect was observed. A more complex effect was recorded for the *M. longifolia* L. and *T. serpyllum* L. extracts. On a high-sugar diet, all the extracts were toxic. All the plant extracts in tested concentrations influenced the DEC, suggesting an impact on gene expression. This study contributes to the expanding knowledge about the beneficial and toxic effects of local medicinal plants, suggesting the need for future studies to elucidate the appropriate use of natural products in therapy.

## 1. Introduction

Medicinal plants have been used since ancient times for their healing properties [1]. Nowadays, the world’s population is still interested in the use of traditional medicine to prevent or alleviate diseases [2]. Research in the field of medicinal plants is constantly developing, as the transition from traditional use to the integration of plant-based therapies into modern health care requires knowledge of the composition of herbal remedies and evidence of the efficacy and safety of these products [3].

A variety of phytochemicals with antioxidant potential are found in fruits, vegetables, grains, and medicinal plants. They are known to protect against chronic diseases due to their ability to neutralize oxidants [4]. Oxidative stress is a body condition characterized by an imbalance between free radicals and antioxidants. Free radicals are highly reactive chemical species with unpaired electrons. Above a limit, they can induce damage to other structures, such as proteins, lipids, and nucleic acids, leading to neurodegenerative and cardiovascular disorders, inflammation, diabetes mellitus, and cancer [5].

*Achillea millefolium* L. (yarrow) is a member of the Asteraceae family, commonly known for its applications in folk medicine [6]. In West Azerbaijan, a dry flower infusion is used against dyspepsia, anorexia, gastralgia, and gynecological, among other complaints [7]. *A. millefolium* L. has been known since the time of the Dacians, who used this plant for its antiviral, anti-inflammatory, analgesic, sedative, cicatrizing, anti-asthmatic, antitussive, appetite stimulant, and detoxifying effect. This plant was considered a remedy for hemorrhoids, digestive, gallbladder, and liver diseases [8]. In Lithuanian traditional medicine, the leaves of *A. millefolium* L. are used externally, fresh or crushed, for wound healing [9]. In Polish folk medicine, the plant is also mentioned for the treatment of open cut wounds [10]. Yarrow aerial parts are used in Europe and Asia to alleviate skin disorders. Lotions and ointments are recommended for external use [6]. This plant contain phenolic compounds, such as caffeic [11,12,13,14], chlorogenic [11,13,14,15,16], ferulic [13,14], and gallic [13] acids, as well as quercetin [11,12,13,15] and rutin [11,12,13,15]. Recent studies have demonstrated that *A. millefolium* L. extracts are potential antioxidant agents [14,17,18,19,20,21]. Anti-inflammatory [22], antimicrobial [23,24], and anticancer [25] effects were also reported for yarrow extracts.

*Mentha longifolia* (L.) Huds. (wild mint) is a medicinal plant belonging to the Lamiaceae family [26]. The leaves of the plant are used in Jordanian traditional medicine as an infusion against constipation, common cold, fever, and general weakness [27]. In India and Iran, *M. longifolia* L. infusions and decoctions from leaves are used for their antiseptic and carminative effect. The wild mint leaf infusion and decoction are also used in Turkey for their carminative effect. Infusion or decoction prepared from wild mint leaves is a popular remedy for gastrointestinal disorders in the folk medicine of India, Iran, Iraq, and Pakistan. The plant is also known by many cultures for alleviating respiratory disorders, such as sore throat (Iraq, Turkey, and Nepal), nasal decongestant (Iraq and Turkey), antitussive, and expectorant (China) [28]. Wild mint contains phenolic compounds, including caffeic [29,30,31,32,33], chlorogenic [29,33], cinnamic [32], ferulic [29,30,32], gallic [30], and syringic [30,31] acids, as well as rutin [29,30,31,32,34] and quercetin [30,31,34]. Some of the plant beneficial health properties have been demonstrated, such as antioxidant [29,30,35,36], antimicrobial [30,35,37], antithrombotic [38], antiproliferative [37], hepatoprotective, and renoprotective [32] effects. 

*Thymus serpyllum* L. (wild thyme) is also a member of the Lamiaceae family, being widely used in traditional medicine against respiratory and digestive complaints [39]. The plant aerial part is used in Bulgarian traditional medicine as a decoction to alleviate cold, stimulate appetite, and for its tranquilizer effect [40]. *T. serpyllum* L. aerial parts are used as a tea in Albania and Kosovo for sedative, neurorelaxant, immunostimulant, anti-asthmatic, carminative, and spasmolytic effects. It is recommended in traditional medicine to alleviate influenza, respiratory inflammations, bronchitis, gastrointestinal disorders, and to improve blood circulation [41]. In Bosnia and Herzegovina, infusion of wild thyme aerial parts is also used to treat digestive and respiratory ailments [42]. Phenolic compounds, such as caffeic [43], chlorogenic [43,44], gallic [44,45], and ferulic [43] acids, as well as catechin [43], rutin [43,45], and quercetin [43], are among the most important secondary metabolites. *T. serpyllum* L. extracts exert various pharmacological effects, including antioxidant [45,46,47,48,49], antimicrobial [46,48,49], and spasmolytic [49].

Polyphenols are generally considered to be safe when they are consumed by healthy individuals as a component of a normal diet. However, some polyphenols have been incriminated for their adverse effects and harmful interactions, especially when they are consumed in high doses [50]. For example, gallic acid has been reported to induce cerebral hemorrhage and muscular hemorrhagic liposis [51], as well as renal, cardiac, and lung injuries [52]. Caffeic acid exerted reproductive and development toxicity in mice by affecting the implantation of the embryos and fetal weight gain [53]. Rutin dose-dependently increased the cardiac toxicity induced by isoprenaline in rats, the toxic effect being attributed to the pro-oxidative effect [54]. Resveratrol has been reported to induced renal toxicity in rats [55]. Cinnamic acid has relatively low toxicity at conventional doses. However, it can produce allergic reactions [56]. Chlorogenic acid can cause hepatotoxicity when used in high doses [57]. Regarding interactions, polyphenols interact with iron supplements used in the treatment of anemia. They act as inhibitors of cytochrome P450 enzymes, causing harmful effects, especially when administered simultaneously with drugs with a narrow therapeutic index, including digoxin, warfarin, and cyclosporine A [50]. Flavonoids interact with anticoagulant drugs resulting in an increased risk of bleeding [58]. Therefore, assessing the toxicity of plant extracts is a mandatory step which involves establishing safe doses, detecting possible interactions and contraindications before introducing plant products into clinical use [59]. 

*Drosophila melanogaster* (fruit fly) is a translational model organism used for a long time in developmental and genetic studies. Compared to mammalians, it has a short lifespan and high reproductive rate, offering the possibility to generate large populations in a short time, which make the fruit fly a valuable candidate for longevity and lifespan studies [60]. Fruit flies are small, easy to handle, cost-effective, and they can be used in studies with fewer ethical issues [61]. Moreover, changes generated by exposure to foods and natural products can be easily observed [62]. Also, the biological, physiological, and metabolic processes of *D. melanogaster* are similar to those of mammalians [60]. 

With this background, the aim of this study is to assess extracts from *A. millefolium* L., *M. longifolia* L., and *T. serpyllum* L., three plant species growing wild in the central Romanian mountainous region, regarding their dual beneficial and toxic potential. In addition, their phenolic profile, total phenolic content, antioxidant activity, and the impact on *D. melanogaster* viability and gene expression by position-effect variegation (PEV) studies were evaluated. To the best of our knowledge this is the first study investigating the effect on viability and PEV in the *D. melanogaster* model system for the extracts from *A. millefolium* L., *M. longifolia* L., and *T. serpyllum* collected from Romania.

## 2. Results and Discussions

### 2.1. Phenolic Compounds Identification and Quantification

The identification and quantification of phenolic compounds in plant extracts was of interest in order to compare which composition impacts their biological activities. Table 1 presents the phenolic profile of the extracts analyzed in this study. The results show that rutin was the main compound found in two of the three hydro-methanolic extracts (MLmet-1 and TSmet-1), while it was the second most abundant in AMmet-1, after (+)-catechin. (+)-Catechin was only detected in AMmet-1. TSmet-1 had a higher rutin content (3183.42 μg/g dw) compared to MLmet-1 (2725.58 μg/g dw) and AMmet-1 (1304.45 μg/g dw). For each plant species, the hydro-methanolic extracts contained higher amounts of rutin than the hydro-ethanolic extracts. AMmet-1 contained about 2.80-fold greater rutin content than AMeth-2. A similar pattern was observed in the case of MLmet-1 and MLeth-2. TSmet-1 had about 1.72-fold higher rutin content than TSeth-2. The most abundant compounds in the hydro-ethanolic extracts were resveratrol in the AMeth-2 (1493.86 μg/g dw), quercetin in MLeth-2 (2077.26 μg/g dw), and cinnamic acid in TSeth-2 (4912.66 μg/g dw). The second most abundant compounds were quercetin in MLmet-1 (883.11 μg/g dw) and TSmet-1 (56.37 μg/g dw), rutin in AMeth-2 (466.88 μg/g dw) and TSeth-2 (1847.72 μg/g dw), and chlorogenic acid in MLeth-2 (1619.75 μg/g dw). High quantities of rutin were also detected in MLeth-2 (973.14 μg/g dw). Some phenolic compounds were detected in concentrations over 100 μg/g dw, such as cinnamic acid in AMeth-2, MLmet-1, and MLeth-2, caffeic acid in TSeth-2, chlorogenic acid in AMmet-1 and AMeth-2, ferulic acid in AMeth-2 and MLeth-2, quercetin and resveratrol in AMmet-1 and TSeth-2. Other phenolic compounds were detected in lower quantities.

Generally, using 70% ethanol as a solvent led to a better recovery of individual phenolic compounds compared to 70% methanol. However, the total quantity of phenolic compounds was higher in the hydro-methanolic extracts because some of them were extracted more efficiently with 70% methanol, such as gallic acid from *A. millefolium* L., syringic acid from *M. longifolia* L. and *T. serpyllum* L., caffeic acid from *A. millefolium* L., rutin from all investigated plant species, and quercetin from *A. millefolium* L. For most of the plant species, if a compound was not detected in the hydro-methanolic extract, it was not detected in the hydro-ethanolic extract either, such as gallic acid in *M. longifolia* L. and *T. serpyllum* L., syringic acid in *A. millefolium* L., chlorogenic acid in *T. serpyllum* L., and (+)-catechin in *M. longifolia* L. and *T. serpyllum* L. Some phenolic compounds were identified only in the hydro-methanolic extracts, such as syringic acid from *M. longifolia* L. and *T. serpyllum* L., caffeic acid and (+)-catechin from *A. millefolium* L. However, in the case of chlorogenic acid extracted from *M. longifolium* L., an opposite pattern was observed. Specifically, it was only detected in the hydro-ethanolic extract and it was not identified in the hydro-methanolic extract.

The level of antioxidants, expressed in terms of the TPC, depends on the plant species and botanical family. The Lamiaceae family generally exhibit a higher TPC and stronger antioxidant activity compared to many, though not all, members of the Asteraceae family [63,64]. 

Comparing the flavonoid profiles of the Asteraceae and Lamiaceae families reveals distinct differences in their chemical composition, structural diversity, and total content, although both are rich in bioactive phenolic compounds. The Asteraceae family is characterized by high structural diversity, with a predominance of flavonols, such as quercetin [65]. 

Resveratrol is a stilbenoid typically associated with grapes and berries, not commonly found in high concentrations in the Asteraceae or Lamiaceae families [66,67,68].

The results of this study are consistent with those of other studies which have reported various amounts of chlorogenic acid [15,23,69], rutin [15,23], and quercetin [15] in the extracts of *A. millefolium* L. collected from Romania. Table S1 (Supplementary Materials) presents a summary of the results of other studies on selected phenolic compounds from the analyzed plant species.

The results of the present study are in agreement with those of the other studies from the specialized literature (presented in Table S1—Supplementary Materials), which have reported that the *A. millefolium* L. extracts contained between 17.46 and 460 μg caffeic acid/g dw [11,16,18,23], 10 and 28,123.17 μg chlorogenic acid/g dw [15,16,18,23,69], and 29 and 2634.46 μg rutin/g dw [15,16,18,23]. Compared to the results obtained in this study, lower quantities of quercetin (14–27.61 μg/g dw) were reported by other researchers [15,16,18]. In contrast with the present study, a total extract obtained from a 70% ethanolic extract and a hydrolyzed extract of *A. millefolium* L. (whole plant) collected from Transylvania did not contain ferulic acid [23]. However, ferulic acid was quantified in a 70% methanolic extract of *Millefolii herba* collected from Poland [16].

Regarding the *M. longifolia* L. extracts, previous studies have reported various quantities of individual phenolic compounds (Table S1—Supplementary Materials). The results of the present study are consistent with those of the other studies that found caffeic acid between less than 2 (or even not detected) and 1540 μg/g dw [29,31,34], rutin between 8.22 and 7571 μg/g μg/g dw [29,31,34], and quercetin between 134.3 and 3180 μg/g dw [31,34]. The *M. longifolia* L. extracts analyzed in the present study contained higher ferulic acid concentrations than that obtained in some other studies, which have reported between less than 2 (or even not detected) and 10.45 μg/g dry [29,31,34]. The results of the present study are in accordance with those in which gallic acid was not detected in the *M. longifolia* L. extracts [31,34]. However, this compound was quantified in the *M. longifolia* L. extract obtained by another extraction method [31]. Similar to our results, some of the other investigated extracts of *M. longifolia* L. contained low (30 μg/g dw) [31] or lacked syringic acid [31,34]. In another extract of *M. longifolia* L., syringic acid was found in a higher concentration (220 μg/g dw) [31].

The phenolic profile of the *T. serpyllum* L. extracts also varied in the studies identified in the specialized literature (Table S1—Supplementary Materials). The content of caffeic acid from the *T. serpyllum* L. hydro-ethanolic extract analyzed in the present study was similar with the results obtained in the other studies [43,45]. The *T. serpyllum* L. extracts assessed in the present study contained higher quantities of rutin and quercetin as compared to the extract of *T. serpyllum* L. collected from Târgu Mureș, Romania [43]. Our findings are in accordance with the results of the other studies which indicated that the *T. serpyllum* L. extracts contain low amounts of ferulic acid [43]. In contrast with the findings of the present study, in which catechin and chlorogenic acid were not detected, other wild thyme extracts contained low quantities of these compounds [43,44]. Other studies found in the literature show a concentration ranging from 50 to 1402 μg/g dw gallic acid [44,45], but this compound was not identified in the *T. serpyllum* extracts analyzed in the present study. The differences in the phytochemical composition or biological activity of the analyzed extracts compared to the data reported in the scientific literature can vary due to several factors, such as the plant part analyzed, its geographical origin, pedoclimatic conditions and altitude, developmental stage, time of harvest, post-harvest processing and storage conditions, exposure to stress, specific extraction parameters (method, solvent, time, temperature, particle size, etc.), and analytical methods used [11,70,71,72,73,74]. Therefore, the observed differences cannot be attributed to a single contributing factor, and further targeted studies are necessary to determine the exact causes. 

Many parameters, such as plant part, polarity of the solvents used for extraction, extraction method, identification and quantification techniques, and environmental conditions influence the composition of plant extracts [30].

The biological effects previously reported for the extracts obtained from these three plant species (*A. millefolium* L., *M. longifolia* L., and *T. serpyllum* L.), as well as their therapeutic applications, including those in traditional medicine, might be supported by the pharmacological effects of some of their most abundant bioactive compounds already described in the literature alongside other less abundant ones. Rutin and resveratrol, the most abundant compounds from the *A. millefolium* L. extracts investigated in this study, were previously found to possess antiviral [75,76,77,78,79,80], anti-asthmatic [81,82], anti-inflammatory [80,83,84], and wound healing [85,86] properties. Other studies revealed the beneficial effects of rutin and resveratrol in alleviating digestive [87,88] and respiratory disorders [89,90,91]. Rutin was the most abundant compound also identified in the MLmet-1 and TSmet-1 extracts, and the above mentioned effects support the applications of these plant species in traditional medicine. Quercetin, the predominant compound from MLeth-2, was reported to exert antiviral [92] and prokinetic [93] effects. Sedative [94] and immunomodulatory [95] properties were previously reported for cinnamic acid, the most abundant compound from TSeth-2.

Therefore, the pharmacological activities of these extracts, driven by their specific phytochemical profiles, can provide a scientific basis for their traditional medicinal use.

### 2.2. Total Phenolic Content and Antioxidant Activity

The results demonstrated that the hydro-methanolic extracts contained a significantly higher total phenolic content (TPC) than the hydro-ethanolic extracts. In both types of extracts, *M. longifolia* L. had the highest TPC, followed by *T. serpyllum* L. and *A. millefolium* L. A comparable TPC was obtained for TSmet-1 and MLeth-2 (Table 2). For both types of extracts, *M. longifolia* L. demonstrated the strongest DPPH radical scavenging activity, followed by *A. millefolium* L. and *T. serpyllum* L. However, only AMeth-2 demonstrated a significantly greater DPPH inhibition compared to AMmet-1, whereas no significant difference was obtained between the hydro-methanolic and hydro-ethanolic extracts of *M. longifolia* L. and *T. serpyllum* L., respectively. Similar rankings of the antioxidant activities were observed for the investigated extracts, both in the ABTS and FRAP assays. Ascorbic acid exerted a higher antioxidant activity compared to the analyzed plant extracts in all applied tests.

A summary of the results of the previous studies regarding the TPC and antioxidant activity of the *A. millefolium* L., *M. longifolia* L., and *T. serpyllum* L. extracts is presented in Table S2 (Supplementary Materials). The results of the present study are in accordance with those previously reported in the specialized literature regarding the TPC and antioxidant activity of the *A. millefolium* L. extracts. Specifically, the studies selected from the specialized literature (Table S2—Supplementary Materials) show that the *A. millefolium* L. extracts have a variable TPC between 0.761 mg and 194.59 mg GAE/g dw [15,16,19,23,24,25,96,97,98], this range includes the values obtained for AMmet-1 (15.62 ± 0.82 mg GAE/g dw) and AMeth-2 (5.32 ± 0.58 mg GAE/g dw). High variations of the TPC of the *M. longifolia* L. extracts were found in the literature (Table S2—Supplementary Materials) ranging from 5.97 to 219.20 mg GAE/g dw [29,35,36,99]. Therefore, the TPC of the MLmet-1 extract (29.32 ± 1.31 mg GAE/g dw) and the MLeth-2 extract (17.65 ± 0.17 mg GAE/g dw) corroborate the earlier studies. Regarding the *T. serpyllum* L. extracts, the values of the TPC found in the other studies (Table S2—Supplementary Materials) varied between 2.45 and 119.52 mg GAE/g dw [45,48,96,100,101,102]. The results obtained for TSmet-1 (19.09 ± 0.61 mg GAE/g dw) and TSeth-2 (11.29 ± 0.37 mg GAE/g dw) are within this range.

Similar to our results, other researchers have reported a higher DPPH radical scavenging activity of the *A. millefolium* L. extract as compared to the *T. serpyllum* L. extract [96]. The DPPH scavenging activity of AMmet-1 and AMeth-2 are similar with those reported by the other studies (Table S2—Supplementary Materials), which demonstrated that *A. millefolium* L. extracts had a DPPH inhibition capacity between 70.36% and 91% [17,20,97,103]. Comparable ABTS scavenging activity and higher FRAP activity were previously reported for *A. millefolium* L. extracts [18]. The *M. longifolia* L. extracts analyzed in the present study had a better DPPH radical scavenging capacity than that found by the other studies [29,104]. Comparable DPPH radical scavenging activity and higher FRAP and ABTS values was previously reported for *T. serpyllum* L. extracts [47,101].

The TPC and antioxidant activity of plant extracts are highly variable and they are influenced by many factors, including the geographic region of the plant species, plant part, solvent used for extraction, plant/solvent ratio, particle size, and time of extraction [36,71,100,102].

### 2.3. Pearson Correlation Analysis Between Total Phenolic Content and Antioxidant Activity

**Figure 1:**
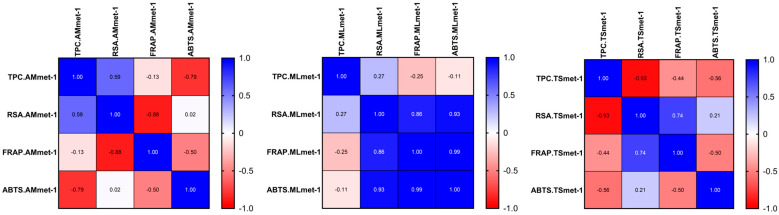
Pearson correlation between the total phenolic content (TPC) and antioxidant activities (RSA, FRAP, and ABTS) in the hydro-methanolic extracts (AMmet-1: *Achillea millefolium* L. hydro-methanolic extract; MLmet-1: *Mentha longifolia* L. hydro-methanolic extract; TSmet-1: *Thymus serpyllum* L. hydro-methanolic extract).

**Figure 2:**
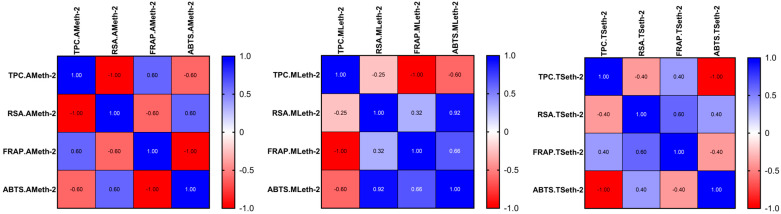
Pearson correlation between the total phenolic content (TPC) and antioxidant activities (RSA, FRAP, and ABTS) in the hydro-ethanolic extracts (AMeth-2: *Achillea millefolium* L. hydro-ethanolic extract; MLeth-2: *Mentha longifolia* L. hydro-ethanolic extract; TSeth-2: *Thymus serpyllum* L. hydro-ethanolic extract).

A Pearson correlation analysis was performed to evaluate the relationships between the TPC and antioxidant activities assessed by RSA, FRAP, and ABTS assays across the investigated plant extracts (*A. millefolium* L., *M. longifolia* L., and *T. serpyllum* L.), obtained using hydro-methanolic and hydro-ethanolic solvents (Figure 1 and Figure 2).

The analysis revealed highly variable and extract-dependent correlation patterns, with both positive and negative associations observed between the TPC and antioxidant activity. The Pearson correlation coefficients ranged from −1.00 to 0.60, indicating substantial variability in both the magnitude and direction of these relationships depending on the plant species and the extraction solvent. However, none of these correlations reached statistical significance (*p* > 0.05), indicating the absence of a consistent linear relationship between the TPC and antioxidant capacity.

A more detailed evaluation showed that the relationship between the TPC and RSA was inconsistent across the extracts. AMmet-1 exhibited a moderate positive correlation (r = 0.59), whereas TSmet-1 showed a strong negative correlation (r = −0.93), and AMeth-2 presented an extreme negative association (r = −1.00). Similarly, the TPC–FRAP and TPC–ABTS relationships varied markedly across the samples, with both positive and negative coefficients observed (TPC–ABTS in AMmet-1: r = −0.79; TPC–FRAP in MLeth-2: r = −1.00; TPC–ABTS in TSeth-2: r = −1.00). These findings clearly demonstrate that the relationship between the TPC and antioxidant activity is not only weak but inconsistent in direction, indicating that the TPC alone is not a reliable predictor of antioxidant capacity in the analyzed extracts. By contrast, strong positive correlations were consistently observed among the antioxidant assays, particularly between ABTS and FRAP (r = 0.99) and between RSA and ABTS (r = 0.93), especially in MLmet-1 and MLeth-2. This suggests a high level of agreement between these methods in evaluating antioxidant potential, despite their different underlying mechanisms.

The lack of significant and consistent correlations between the TPC and antioxidant activity may be explained by several factors. First, the TPC represents a global quantitative parameter and does not reflect the qualitative composition of phenolic compounds, which may differ substantially in antioxidant efficiency [105]. Second, other bioactive constituents, such as flavonoids, terpenoids, or vitamins, may contribute significantly to the overall antioxidant activity [106]. Third, the different mechanisms underlying the assays—radical scavenging (RSA, ABTS) and reducing power (FRAP)—may result in distinct responses depending on the phytochemical profile of each extract [107].

Overall, the results indicate that antioxidant activity is governed by a complex interplay of phytochemical constituents and cannot be reliably predicted based solely on the TPC.

### 2.4. The Impact of Plant Extracts on Drosophila Melanogaster Viability

#### 2.4.1. Neutral Diet

*Drosophila melanogaster* is one of the most suitable model organisms used for a developmental toxicity evaluation of plants [62]. For this purpose, we investigated the influence of the concentration of the AMmet-3, MLmet-3, and TSmet-3 extracts on the viability and developmental time of the fruit fly reared on three culture media (0M, ND, and HSD). It is worth noting that all individuals tested had the same age, genotypes, and epigenomes, and all the differences between the control and tested groups could be attributed to the plant extracts [62]. 

The life cycle of *D. melanogaster* was previously monitored. The 0–2-hour-old embryos transform into first-instar larvae after 24 h. If there are sufficient nutrients, the first-instar larvae develop into the second and third instar larvae, each stage lasting 24 h. After the completion of the third-instar larval stage (three days), pupation occurs. Metamorphosis takes place in the next four to five days, which ends with the hatching of adult flies. On the other hand, if nutrients are insufficient to support a stage of development, the life cycle will be interrupted and the individuals will die without reaching the next stage of life [108].

Considering day 0 the day of seeding embryos into the medium, for all the extracts at the concentration of 100 μL of extract/2 mL of 0M, larvae were observed on day 2. Some of them died by day 10 and the majority were dead by day 15. All of the larvae were dead by day 19. At the concentration of 500 μL/2 mL of 0M, larvae emerged on day 3. By day 13, most of them were dead. For the concentration of 1 mL of extract/2 mL of 0M, some small larvae were seen on day 6. No pupae or flies were observed. Since 0M was free of nutrients, these results suggest that AMmet-3, MLmet-3, and TSmet-3 did not contain enough nutrients to support the completion of the larval stage of *D. melanogaster*.

#### 2.4.2. Normal Diet

**Figure 3:**
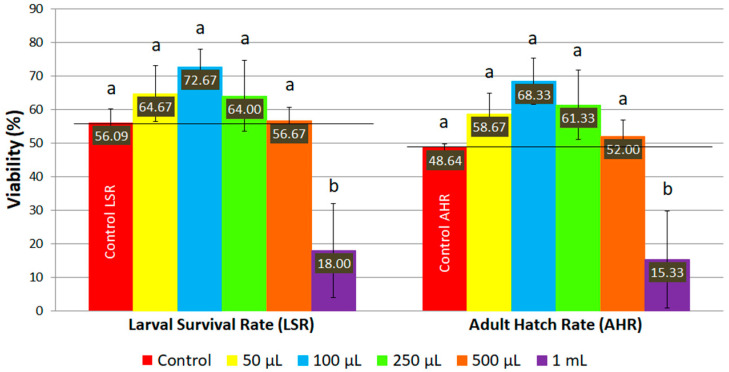
The viability of *D. melanogaster* on ND supplemented with AMmet-3. Different letters (a, b) indicate a significant difference between the mean values (*p* < 0.05).

The viability of *D. melanogaster* fed with ND supplemented with AMmet-3 increased as the concentration of plant extract increased up to 100 μL per 2 mL of ND, where maximum survival rates were reached both for larvae (72.67%) and adult individuals (68.33%) (Figure 3). For higher concentrations (0.25, 0.50, and 1 mL of AMmet-3 per 2 mL of ND), the viability of *D. melanogaster* decreased as the concentration of the extract increased. However, at concentrations of 0.05, 0.10, 0.25, and 0.50 mL of extract per 2 mL of ND, higher survival rates of larvae and adult flies were recorded compared to the control group, but the mean values were not significantly different. At 1 mL of AMmet-3 per 2 mL of ND, a significant decrease of the viability was recorded compared to the control group.

**Figure 4:**
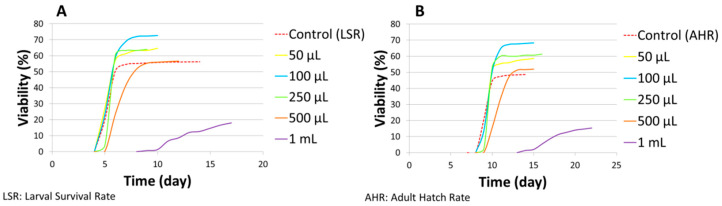
The developmental time of *D. melanogaster* on ND supplemented with AMmet-3.

Simultaneously, the developmental time of *D. melanogaster* raised on ND supplemented with AMmet-3 was recorded (Figure 4). No differences were observed in the developmental time of larvae between the control and the groups treated with AMmet-3 at concentrations from 50 to 500 μL of AMmet-3 per 2 mL of ND (Figure 4A). Compared to the control, at 1 mL of AMmet-3 per 2 mL of ND, the larval period was extended by four days, with a significant decrease of the larval survival rate. A one-day delay was observed in the development of pupae to adult individuals in the groups treated with AMmet-3 at 50–500 μL/2 mL concentration range as compared to the control group, without affecting the viability (Figure 4B). At the concentration of 1 mL AMmet-3 per 2 mL ND, the development of pupae to adult individuals was extended by five days, with a significant decrease of the adult hatch rate.

Hormesis is a biphasic dose response, with beneficial or stimulatory effects at low doses and inhibition or toxicity at high doses. Some phytocompounds in low doses can exert a hormetic effect, which is an adaptive response of an organism to moderate stress [109]. The in vivo *D. melanogaster*-based viability assay revealed that the AMmet-3 extract tends to exert a hormetic effect with increasing the survival rates at low doses and inducing toxicity at high doses. 

Previous studies have reported the hormetic effect induced by phytocompounds in *D. melanogaster*. Rutin, one of the most abundant compound detected in the AMmet-3 extract, extended the lifespan of fruit flies through a hormetic effect at a 100–800 μM concentration range. Furthermore, it significantly increased the percentage of pupation and adult hatching at 200 and 400 μM [110]. 

In another study, diet supplementation with resveratrol at 200 μM significantly increased the viability of pre-adult individuals according to the control, while at lower (25–100 μM) and higher (800 μM) concentrations no significant differences were reported compared to the control. Resveratrol did not significantly impact the developmental time of the pre-adults. However, supplementation of the larval diet with resveratrol extended the lifespan of adult individuals by stimulating the activity of the antioxidant enzymes which are involved in reactive oxygen species scavenging [111]. A previous study reported that resveratrol at low and moderate doses (7.5–60 mg/kg diet) increased the lifespan of fruit flies in a dose-dependent manner by 41.9% as compared to the control. However, at higher doses (120 mg/kg) no extinction of the lifespan was observed, suggesting a hormetic-like effect [112]. Previous research has highlighted the influence of the sex and the nutrient content of the diet. A standard diet supplemented with resveratrol up to 200 μM did not influence the lifespan of *D. melanogaster* males and females. Unlike males, an increase in lifespan was observed in females in the group fed a low-sugar, high-protein diet and 200 μM resveratrol and in the group fed a high-fat diet supplemented with 400 μM resveratrol [113].

Another study demonstrated that a normal diet supplemented with 50 and 100 μM gallic acid could reduce the oxidative stress in a *D. melanogaster* model of Alzheimer’s disease. Specifically, it decreased the reactive oxygen species and malondialdehyde levels and increased the catalase activity and total thiol level [114]. Quercetin, another bioactive compound identified in the investigated extracts, could also influence the antioxidant parameters in fruit flies. A previous study reported that the supplementation of the flies’ diet with quercetin (100 and 200 mg/g) increased the catalase activity, and a higher concentration of quercetin (300 mg/g) decreased the malondialdehyde level [115].

Comparable results were reported by Uysal et al. (2007) [116], who investigated the toxicity of *A. millefolium* L. (flowers and leaves) water extract at 1, 5, and 10 mL/100 mL standard food medium against *D. melanogaster*. They observed that the plant extract did not impact the duration of metamorphosis, since adult individuals emerged on the 9th day and lasted until the 17th day, both for control and test groups. Similar to our results, they reported that the viability of the flies increased in a concentration-dependent manner, without being significantly different according to the control. They mentioned that the observed effect could be attributed to the antioxidant effect of the extract [116]. A similar biphasic effect with stimulation at low doses and inhibition at high doses was observed in our previous study that assessed the impact of *Lythrum salicaria* L. extract on the viability of fruit flies [108]. Similar to our results, santol flesh extract exhibited a biphasic effect regarding adult emergence rate, with a maximum at 60 mg/mL and lower values at 20, 40, 80, and 100 mg/mL [117].

**Figure 5:**
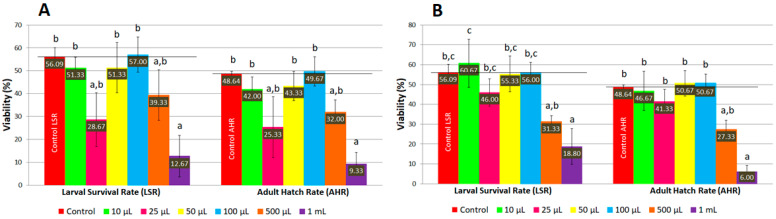
The viability of *D. melanogaster* on ND supplemented with MLmet-3 (**A**) and TSmet-3 (**B**). Different letters (a, b, c) indicate a significant difference between the mean values (*p* < 0.05).

In the case of ND supplemented with MLmet-3 and TSmet-3, a similar pattern was observed regarding the viability of *D. melanogaster* (Figure 5). Specifically, for the concentrations of 10 and 25 μL of extract per 2 mL of ND, the viability of *D. melanogaster* decreased as the extract concentration increased. At these two concentrations, a higher viability compared to the control was only observed for the larvae in the group treated with 10 μL of TSmet-3 per 2 mL of ND. However, no significant difference was detected between the control and the tested groups at the above-mentioned concentrations. The viability increased in a dose-dependent manner at a concentration range of 25–100 μL of extract per 2 mL of ND, followed by a decrease of the viability at 0.1–1 mL concentration range.

**Figure 6:**
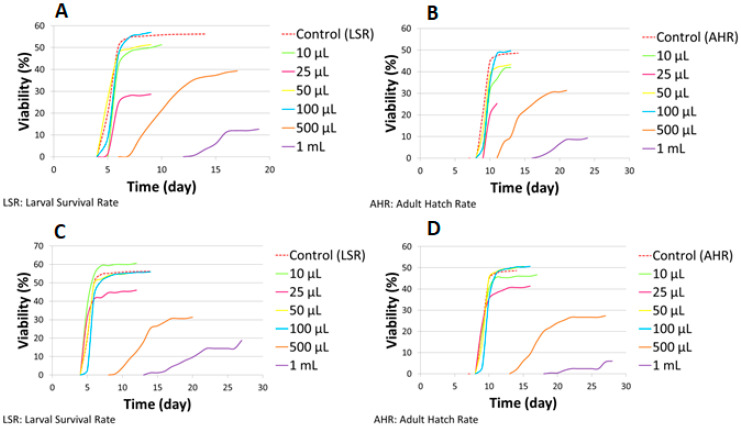
The developmental time of *D. melanogaster* on ND supplemented with MLmet-3 (**A**,**B**) and TSmet-3 (**C**,**D**).

Regarding larval developmental time, no differences were recorded with respect to the control in the groups treated with MLmet-3 and TSmet-3 at 10–100 μL/2 mL ND, except the group treated with 10 μL of MLmet-3 per 2 mL of ND, in which one day extinction was observed (Figure 6A,C). The larval stage was prolonged by two and eight days at 0.5 and 1 mL of MLmet-3 per 2 mL of ND. For 0.5 and 1 mL of TSmet-3 per 2 mL of ND, the larval period was extended by four and nine days compared to the control. The pupal stage extension varied depending on the extract concentration, between 1 and 9 days in the case of MLmet-3 and 1 and 11 days in the case of TSmet-3 (Figure 6B,D).

In the present study, MLmet-3 and TSmet-3 exerted a triphasic dose–response, with inhibition at ultra-low concentrations, stimulation at low concentrations, and inhibition at high concentrations [118]. The triphasic dose–response relationship implies a reparative response that occurs after the threshold for detecting damage is exceeded. Specifically, very low concentrations of a toxicant can induce moderate damage without being detected by the biological system. At low doses, more damage can occur, exceeding the threshold for detecting damage. At moderate concentrations, damage can be detected, and the system will initiate a reparative response (the hormetic domain of the triphasic dose–response curve). At higher concentrations, more damage occurs, and the reparative process will be overloaded, resulting in massive damage [119].

#### 2.4.3. High-Sugar Diet

**Figure 7:**
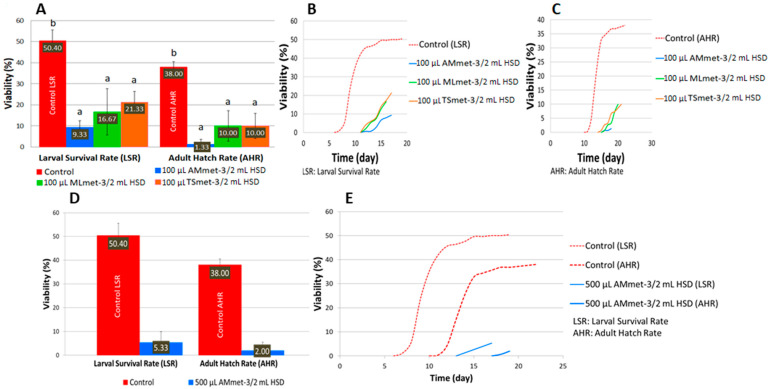
The viability (**A**,**D**) and developmental time (**B**,**C**,**E**) of *D. melanogaster* on a HSD supplemented with plant extracts. Different letters (a, b) indicate a significant difference between the mean values (*p* < 0.05).

Compared to the ND, the HSD decreased the larval and adult hatch rates of the controls simultaneously with a developmental shift. Supplementation of the HSD with the investigated plant extracts (AMmet-3, MLmet-3, and TSmet-3) increased the toxicity compared to the supplemented ND. At the concentration of 100 μL of extract/2 mL of HSD, all the extracts significantly decreased the viability of the larvae and pupae compared to the control; AMmet-3 was the most toxic, followed by MLmet-3 and TSmet-3 (Figure 7A). At 500 μL of extract/2 mL of HSD, MLmet-3 and TSmet-3 were completely toxic, while AMmet-3 reduced the larval survival and adult hatch rates at 5.33% and 2.00%, respectively (Figure 7D). At 1 mL of extract/2 mL of HSD, no larvae or adult individuals were detected. Overall, the toxicity of the HSD supplemented with the tested extract concentrations increased in a dose-dependent manner. Moreover, the decrease in viability is accompanied by a change in the development time, with a delay of approximately 5 days at 100 μL of extract/2 mL of HSD (Figure 7B,C) and 7 days at 500 μL of AMmet-3 per 2 mL of HSD (Figure 7E).

This study is in agreement with previous reports where a HSD also decreased the survival rate of *D. melanogaster* compared to a normal diet [120,121]. Furthermore, other researchers have also observed a developmental delay in larval or pupal stages after the exposure of *D. melanogaster* to a HSD [121,122,123]. Feeding *D. melanogaster* on a HSD has been previously reported to induce a type 2 diabetes mellitus-like phenotype by increasing glucose and triglycerides levels [120,121,122,123], stimulating the activity of carbohydrate hydrolyzing enzymes, such as α-amylase and α-glucosidase [120,122], modulating the expression of genes implicated in the carbohydrate metabolism [122,123,124], and reducing the body size, body weight, and movement capacity of the larvae [124]. It was also associated with insulin resistance and obesity [123]. Moreover, a HSD was reported to generate oxidative stress [120,121] by increasing the level of reactive oxygen species and thiobarbituric acid reactive species, and by decreasing the level of thiol, glutathione-S-transferase, superoxide dismutase, and catalase. Cognitive decline and brain damage could be the consequences of a HSD through increased acetylcholinesterase and monoamine oxidase activity [120].

Other researchers have demonstrated the antidiabetic potential of plant products in *Drosophila melanogaster* model, such as bayberry leaves [122], *Flos Chrysanthemi Indici* extract [125], *Cordia myxa* [124], *Syzygium cumini* [121], and moringa leaves [120]. On the other hand, some plant extracts were not effective in ameliorating the harmful effects induced by a HSD, some of them even exacerbating the toxicity. Similar to our results, *Syzygium malaccense* extract has shown a dose-dependent toxic effect. At concentrations of 0.625–2.5%, the survival rate of larvae and pupae decreased (significantly, except for the viability of pupae at the concentration of 0.625%). At the concentration of 0.625%, a low toxicity was reported without major developmental issues; but, at higher concentrations (1.25-2.5%), mortality increased and the development was delayed [124]. Similar results were observed for *Lythrum salicaria* L. extract, which exacerbated the toxic effect of the HSD simultaneously with a developmental shift [108]. Overall, these results suggest that an investigation of toxicity is crucial for plant extracts before introducing them into therapy as various interactions could occur, resulting in protective or toxic effects, depending on the type of extract and diet.

### 2.5. The Impact of Plant Extracts on Position-Effect Variegation

**Figure 8:**
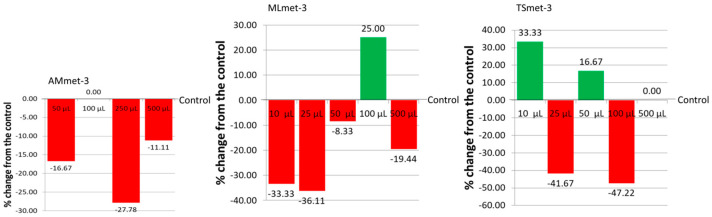
Drosopterin % change from the control in the eyes of *D. melanogaster* fed with ND supplemented with plant extracts.

In order to study the impact of the investigated extracts on PEV, we quantified the red pigment, drosopterin, from the eyes of the *D. melanogaster w^m4h^* strain. The results revealed that all three extracts influenced the expression of genes, leading to different drosopterin content in the eyes of adult male individuals (Figure 8). Compared to the control, three of four tested concentrations (50, 250, and 500 μL per 2 mL of ND) decreased the drosopterin eye content in *D. melanogaster w^m4h^*. No difference compared to the control was recorded at the concentration of 100 μL of AMmet-3 per 2 mL of ND. The concentration of 100 μL of MLmet-3 per 2 mL of ND increased the drosopterin eye content, while the other tested concentrations decreased the drosopterin levels compared to the untreated control. Drosopterin content was elevated in the eyes of male individuals fed with 10 and 50 μL of TSmet-3 per 2 mL of ND, while it was lower than the control at 25 and 100 μL/2 mL of ND. Compared to the control, 500 μL of TSmet-3 per 2 mL of ND had no influence on drosopterin eye content.

PEV is a phenomenon that occurs when a gene located in the euchromatin region is relocated to the heterochromatin region resulting in a variegated phenotype. The genes involved could be stochastically silenced by the spread of heterochromatin across the euchromatin/heterochromatin border [126]. The *white* gene encodes a transporter responsible for red eye pigmentation in wild fruit flies [127]. The *D. melanogaster w^m4h^* strain is an intensively studied model of PEV, in which the *white* gene is relocated in the pericentric heterochromatin zone of the X chromosome [128]. This rearrangement leads to expression of the *white* gene in some ocular cells and inhibition of its expression in other cells, resulting in individuals with white-mottled eyes [126]. The results of this study are consistent with the findings of our previous study, according to which *Lythrum salicaria* L. extract influenced the drosopterin concentration in adult male individuals of *D. melanogaster w^m4h^* compared to the untreated control [108].

## 3. Materials and Methods

### 3.1. Chemical and Reagents

Chloroform, methanol, glycerol, acetic acid, 2,2-diphenyl-1-picrylhydrazyl (DPPH), 2,4,6-tripyridyl-s-triazine (TPTZ), and ammonium hydroxide were acquired from the Sigma-Aldrich Company, St. Louis, MO, USA. Gallic acid, chlorogenic acid, caffeic acid, syringic acid, ferulic acid, cinnamic acid, (+)-catechin, rutin, quercetin, and resveratrol were purchased from Dr. Ehrenstorfer GmbH, Augsburg, Germany. Phosphomolybdotungstic reagent, potassium persulfate, charcoal, saccharose, and agar powder were obtained from VWR Chemicals, Leuven, Belgium. Sodium carbonate, ascorbic acid, sodium acetate, FeCl_3_, and HCl were acquired from Chemical Company S.A., Iași, Romania. 2,2′-azinobis [3-ethylbenzothiazoline-6-sulfonic acid]-diammonium salt (ABTS), trolox, and nipagin were purchased from Thermo Fisher Scientific, Kandel, Germany. Ethanol was obtained from Chimreactiv S.R.L., Bucharest, Romania.

### 3.2. Plant Collection

The aerial parts of the studied medicinal plants were collected from the mountainous region in Sibiu County, Romania, in July 2021 (*Achillea millefolium* L.—45°41′20” N, 23°59′9” E, 1130 m; *Mentha longifolia* L.—45°41′22” N, 23°59′29” E, 1100 m; *Thymus serpyllum* L.—45°42′34” N, 24°1′33” E, 630 m). They were authenticated by the botanist Dr. Oana Danci, according to the “Vascular Plants of Romania, illustrated field guide” by Sârbu et al. (2013) [129]. A voucher specimen of each vegetal product is held at the Faculty of Medicine (Pharmacy specialization), “Lucian Blaga” University of Sibiu, Sibiu, Romania (*A. millefolium* L. no. 101/2, *M. longifolia* L. no. 101/3, and *T. serpyllum* L. 101/4). After harvesting, the raw materials were chopped and shade dried at room temperature. After drying, the vegetal products were kept in labeled paper bags at room temperature, away from light until extraction.

### 3.3. Extraction Procedure

Two types of extracts were prepared from each plant species. The vegetal aerial parts were milled using a domestic coffee mill until a powder was obtained. For the first extraction, the powder/solvent ratio was 0.5:10 *w*/*v*. The powder and the solvent (70% methanol) were introduced in an Erlenmeyer flask with a ground-glass stopper. The extraction was performed in an ultrasonic water bath at 40 °C for 30 min. The selection of the solvent mixture (methanol/distilled water, 70:30, *v*/*v*) was based on a previous study that showed that mixing methanol and water in this ratio extracts a greater amount of phenolic compounds than when using methanol alone [70]. Subsequently, the mixture was cooled, filtered in a 10 mL volumetric flask, and brought to mark with the same solvent. Three distinct extracts, named as *A. millefolium* L. hydro-methanolic extract (AMmet-1), *M. longifolia* L. hydro-methanolic extract (MLmet-1), and *T. serpyllum* L. hydro-methanolic extract (TSmet-1) were obtained. For the second extraction, the powder/solvent (70% ethanol) ratio was 1:10, *w*/*v*. Three different extracts, named as *A. millefolium* L. hydro-ethanolic extract (AMeth-2), *M. longifolia* L. hydro-ethanolic extract (MLeth-2), and *T. serpyllum* L. hydro-ethanolic extract (TSeth-2) were obtained. As soon as the extracts were prepared, their chemical composition and antioxidant capacity were assessed. To avoid the toxicity of methanol in the testes performed on *D. melanogaster*, the hydro-methanolic extracts were further processed by evaporating the methanol. The hydro-methanolic extracts were chosen due to the expectation of a grater phenolic content and a stronger antioxidant capacity compared to the hydro-ethanolic extracts. The products obtained after methanol evaporation were diluted with glycerol in a 2:1, *v*/*v* ratio, resulting the final mixtures labeled as AMmet-3, MLmet-3, and TSmet-3 [108].

### 3.4. HPLC-DAD Analysis

The phenolic profile of the extracts was assessed by an HPLC method adapted after different methods from the specialized literature [34,108,130], using an SCL-40 HPLC system (Shimadzu, Kyoto, Japan) provided with a quaternary pump, autosampler, thermostatted column oven, and photodiode array detector. A Nucleosil C18 column, set to 40 °C, 250 mm × 4.6 mm, and i.d. 5 μm, was used. The injection volume of the sample was 5 μL. The elution was performed in gradient, at a flow rate of 0.8 mL/min, using a mobile phase with two components: (1) purified water/acetic acid 96:4, *v*/*v* and (2) methanol. The change in the composition of the mobile phase over time was set as follows: 85% (1) and 15% (2) for the first 15 min, 75% (1) and 25% (2) for the next 5 min, 15% (1) and 85% (2) for 10 min, 85% (1) and 15% (2) for 5 min, and 85% (1) and 15% (2) for the last min. The detection was performed at 280 nm for gallic acid, (+)-catechin, syringic acid, and cinnamic acid, at 306 nm for resveratrol, at 330 nm for chlorogenic acid, caffeic acid, and ferulic acid, and at 360 nm for rutin and quercetin. Concentrations between 0.2 and 50 μg/mL were tested. All the standards used for the identification and quantification of the phenolic compounds presented purity greater than 94%: purity > 99% for gallic acid, ferulic acid, cinnamic acid, caffeic acid, and resveratrol; > 98% for (+)-catechin; > 95% for syringic acid, chlorogenic acid, and quercetin; > 94% for rutin. They were analyzed due to their potential beneficial properties. All the analyses were performed in triplicate. The results were expressed as μg of compound/g of dry weight.

### 3.5. Spectrophotometric Analysis

#### 3.5.1. Total Phenolic Content

The total phenolic content (TPC) of the extracts was estimated by the Folin–Ciocâlteu method. In a test tube, the extract was mixed with Phosphomolybdotungstic reagent, distilled water, and 290 g/L of sodium carbonate solution in a volume ratio of 0.4:1:15:2. The mixture was shaken for 10 min and kept in a water bath at 40 °C for 20 min. Subsequently, the mixture was cooled at room temperature and its absorbance at 760 nm was measured against a blank using a UV-1900 UV-VIS spectrophotometer (Shimadzu, Kyoto, Japan) [131]. 

For the calibration curve, solutions with concentrations ranging from 0.86 to 8.57 μg gallic acid/mL were prepared. All the analyses were performed in triplicate. The results were expressed as mg gallic acid equivalents (GAE)/g dry weight.

#### 3.5.2. DPPH Radical Scavenging Assay

The DPPH radical scavenging assay was performed to analyze the antioxidant activity of the extracts. Prior to the analysis, DPPH was dissolved in methanol to prepare a 25 μg/mL DPPH stock solution. It was kept in a dark place for 2 h before analysis. A mixture consisting of 1.94 mL DPPH stock solution and 60 μL extract was prepared and kept away from light at room temperature for 15 min, until absorbance stabilization. Subsequently, the absorbance at 515 nm was reached against a blank using a UV-1900 UV-VIS spectrophotometer (Shimadzu, Kyoto, Japan) [71]. A solution of 1 mg ascorbic acid/mL was used as a positive control.

For the calibration curve, solutions with concentrations ranging from 0.5 to 25 μg DPPH/mL were prepared. All the analyses were performed in triplicate. The results were expressed as DPPH inhibition percentages (I%), which were calculated by formula (1):

(1) $$I\%=\frac{C_{st}-C_{sam}}{C_{st}},$$

$C_{st}$ is the concentration of the stock solution and $C_{sam}$ is the concentration of the sample, both in μg DPPH/mL.

The concentration of DPPH in the sample ($C_{sam}$), expressed as μg DPPH/mL, was calculated with Formula (2):

(2) $$C_{sam}=\frac{A_{sam}-intercept}{slope},$$

$A_{sam}$ is the absorbance of the sample, intercept is the intercept of the calibration curve, and slope is the slope of the calibration curve.

#### 3.5.3. ABTS Radical Scavenging Assay

Due to the fact that a single test is insufficient to evaluate the antioxidant capacity of an extract, in addition to the DPPH assay, the ABTS radical scavenging assay was conducted. ABTS and potassium persulfate were dissolved in water to form a 7 mM ABTS and 2.45 mM potassium persulfate stock solution. It was left at room temperature away from light for 16 h prior to analysis. When the time had passed, the stock solution was diluted until the absorbance at 734 nm reached 0.7 ± 0.02. A mixture consisting of 2.5 mL of the stock solution and 25 μL of the extract was prepared in a test tube and vortexed for 30 s. Its absorbance at 734 nm was measured after 1 min, using a UV-1900 UV-VIS spectrophotometer (Shimadzu, Kyoto, Japan) [132]. A solution of 1 mg ascorbic acid/mL was used as a positive control.

For the calibration curve, solutions with concentrations ranging from 0.125 to 2 mmol trolox/L were prepared. All the analyses were performed in triplicate. The results were expressed as mmol trolox equivalents (TE)/g dry weight.

#### 3.5.4. Ferric-Reducing Antioxidant Power (FRAP) Assay

In order to evaluate the antioxidant activity of the extracts, their capacity to reduce Fe^3+^ to Fe^2+^ was investigated by the FRAP assay. Three stock solutions were prepared: 300 mM acetate buffer (pH = 3.6), 20 mM FeCl_3_, and 10 mM TPTZ acidified with 150 μL HCl. The FRAP working solution was obtained just before the analysis by mixing 50 mL of the acetate buffer solution with 5 mL of the FeCl_3_ solution and 5 mL of the TPTZ solution. A mixture consisting of 0.1 mL of the plant extract, 1.5 mL of the FRAP reagent, and 2 mL of distilled water was prepared in a test tube and kept at room temperature protected from light. The absorbance at 595 nm against a blank was recorded using a UV-1900 UV-VIS spectrophotometer (Shimadzu, Kyoto, Japan) [132]. A solution of 1 mg ascorbic acid/mL was used as a positive control.

For the calibration curve, solutions with concentrations ranging from 0.15 to 0.50 μmol trolox/mL were prepared. All the analyses were performed in triplicate. The results were expressed as μmol TE/g dry weight.

### 3.6. In Vivo Drosophila melanogaster Viability Assay

This study investigated the toxicity of the AMmet-3, MLmet-3, and TSmet-3 extracts prepared as presented in the Section 2.3 by using an in vivo test on the *D. melanogaster* model system. The protocol consisted of assessing the viability of *D. melanogaster* fed with three types of diets supplemented with plant extracts at various concentrations. A culture medium with no nutrients (0 M) was prepared by mixing 1 g of charcoal with 100 mL of distilled water and 1 g of agar powder. The distilled water was previously boiled to prevent mold contamination. Subsequently, the charcoal and agar powder were added under continuous stirring, boiled for 30 s, and cooled to 45 °C. The second medium simulated a normal diet (ND) and was obtained by introducing 70 g of yeast paste into 1200 mL of distilled water under continuous stirring, followed by addition of 52 g of saccharose and 30 g of wheat flour. After boiling, 10 g of agar powder were added and the mixture was boiled for 30 min. After cooling to 50 °C, 1 g of nipagin was incorporated to preserve the microbiological stability. The third medium simulated a high sugar diet (HSD). It was prepared similarly to the ND, but with a concentration of saccharose of 0.75M [133]. 

Three extract concentrations were tested on 0M and HSD: 4.76%, 20%, and 33.33%. They were obtained by introducing 0.1, 0.5, and 1 mL of extract in 2 mL of medium dispensed in culture vials. These concentrations were selected based on a previous study that assessed the influence of plant extracts on the viability of *D. melanogaster*, which tested concentrations between 11% and 50% [133]. Based on our observations of 0M, for a better understanding of the dose–effect response, we decided to test additional extract concentrations on ND. For AMmet-3, a total of five concentrations were tested: 2.44%, 4.76%, 11.11%, 20%, and 33.33% (prepared by mixing 0.05, 0.1, 0.25, 0.5, and 1 mL of extract with 2 mL of culture medium). For MLmet-3 and TSmet-3, a total of six extract concentrations were tested on ND: 0.5%, 1.23%, 2.44%, 4.76%, 20%, and 33.33%, (obtained by mixing 0.01, 0.025, 0.05, 0.1, 0.5, and 1 mL of extract with 2 mL of culture medium). 

An embryo collector containing about 200 of 5-day old male and female *D. melanogaster w^m4h^* individuals was placed over a Petri dish filled with 0M supplemented with yeast paste. On the next day, 50 embryos of *D. melanogaster w^m4h^* aged between 0 and 2 hours were introduced into each of the vials containing the media supplemented with plant extracts under the microscope (VWR VisiScope SZB260 stereo microscope, VWR, Milano, Italy) by using a fine forceps. The negative control was prepared by introducing 50 embryos into a vial with 2 mL of culture medium. The number of newly emerged pupae and flies were monitored daily. All the experiments were performed in triplicate, under specific conditions at 25 °C and constant humidity. The results were expressed as percentage of viability [133].

### 3.7. Position Effect Variegation Study

This study evaluated the position-effect variegation by quantifying the drosopterin pigment in the eyes of *D. melanogaster* adult male individuals. For each concentration tested on ND, ten males were randomly selected and their heads were removed with a scalpel under a stereo microscope (VWR VisiScope SZB260 stereo microscope, VWR, Milano, Italy). The pigments were extracted by mixing the heads with 300 μL of chloroform and 300 μL of 0.1% ammonium hydroxide solution, followed by homogenization and centrifugation at 4000 revolutions per min for 4 min. A volume of 200 μL of supernatant was added over 500 μL of 0.1% ammonium hydroxide solution, followed by homogenization. The absorbances were read against ethanol at 485 nm using a UV/Vis Ultrospec 2100 pro spectrophotometer (Biochrom Ltd., Cambridge, UK) [134].

### 3.8. Statistical Analysis

The results of the tests performed in triplicate were expressed as mean values and standard deviation. The significant difference between the mean was calculated by one-way ANOVA (Tukey’s post hoc test) using IMB SPSS statistics for Windows, version 26 (IBM Corp., Armonk, NY, USA). A Pearson correlation analysis was performed using GraphPad Prism 8 (GraphPad Software, San Diego, CA, USA) to evaluate the relationships between total phenolic content and antioxidant activities. A *p*-value < 0.05 was considered statistically significant.

## 4. Conclusions

This study investigated the phenolic profile, antioxidant activity, and the impact on *D. melanogaster* viability and gene expression, of natural extracts obtained from *A. millefolium* L., *M. longifolia* (L.) Huds., and *T. serpyllum* L. Rutin was the main phenolic compound in the *M. longifolia* (L.) Huds. and *T. serpyllum* L. hydro-methanolic extracts, while (+)-catechin predominated in the *A. millefolium* L. hydro-methanolic extract. Resveratrol, quercetin, and cinnamic acid were the most abundant phenolic compounds in the hydro-ethanolic extracts of *A. millefolium* L., *M. longifolia* (L.) Huds., and *T. serpyllum* L., respectively. Due to the different amounts of polyphenols in the extracts, they demonstrated variable antioxidant activity.

Regarding the toxicity, low concentrations (50–500 μL/2 mL normal medium) of the *A. millefolium* L. extract did not affect the viability of *D. melanogaster*, while the concentration of 1 μL/2 mL of the normal medium was toxic. A more complex response was recorded in the case of normal medium supplemented with the *M. longifolia* (L.) Huds. and *T. serpyllum* L. extracts. A high sugar diet increased the toxicity of the investigated extracts.

The drosopterin content was affected by the tested extracts, suggesting the presence of some phytochemicals that could interfere with gene expression. Future studies are needed to clearly define the conditions for using these extracts in therapy.

## Figures and Tables

**Table 1:** Concentration of phenolic compounds in *A. millefolium* L., *M. longifolia* L., and *T. serpyllum* L. extracts.

Compound (μg/g dw)		Extract Type					
		AMmet-1	AMeth-2	MLmet-1	MLeth-2	TSmet-1	TSeth-2
Phenolic acids	Gallic acid	64.75 ± 0.73 ^b^	53.56 ± 2.65 ^c^	n.d. ^a^	n.d. ^a^	n.d. ^a^	n.d. ^a^
	Cinnamic acid	53.79 ± 0.18 ^b^	224.57 ± 0.29 ^d^	152.73 ± 1.30 ^c^	349.03 ± 0.23 ^e^	9.51 ± 0.30 ^a^	4912.66 ± 8.11 ^f^
	Syringic acid	n.d. ^a^	n.d. ^a^	18.35 ± 0.19 ^b^	n.d. ^a^	28.02 ± 0.30 ^c^	n.d. ^a^
	Caffeic acid	18.10 ± 0.04 ^b^	n.d. ^a^	36.19 ± 0.35 ^c^	39.35 ± 0.15 ^d^	42.59 ± 0.21 ^e^	132.11 ± 0.68 ^f^
	Chlorogenic acid	199.40 ± 0.80 ^b^	364.67 ± 1.29 ^c^	n.d. ^a^	1619.75 ± 1.41 ^d^	n.d. ^a^	n.d. ^a^
	Ferulic acid	36.37 ± 0.90 ^c^	223.42 ± 2.22 ^f^	51.67 ± 1.06 ^d^	174.56 ± 0.43 ^e^	3.29 ± 0.36 ^a^ (n.q.)	28.40 ± 0.61 ^b^
Flavonoids	(+)-Catechin	2058.49 ± 61.19 ^b^	n.d. ^a^	n.d. ^a^	n.d. ^a^	n.d. ^a^	n.d. ^a^
	Rutin	1304.45 ± 2.51 ^c^	466.88 ± 1.37 ^a^	2725.58 ± 28.38 ^e^	973.14 ± 0.35 ^a^	3183.42 ± 16.12 ^f^	1847.72 ± 3.13 ^d^
	Quercetin	186.13 ± 0.27 ^c^	64.05 ± 0.29 ^b^	883.11 ± 2.94 ^e^	2077.26 ± 0.80 ^f^	56.37 ± 0.55 ^a^	391.98 ± 0.36 ^d^
Stilbenes	Resveratrol	336.31 ± 0.84 ^e^	1493.86 ± 3.53 ^f^	4.21 ± 0.33 ^a^ (n.q.)	60.65 ± 1.18 ^c^	23.27 ± 4.03 ^b^	230.47 ± 11.34 ^d^

AMmet-1: *Achillea millefolium* L. hydro-methanolic extract; MLmet-1: *Mentha longifolia* L. hydro-methanolic extract; TSmet-1: *Thymus serpyllum* L. hydro-methanolic extract; AMeth-2: *Achillea millefolium* L. hydro-ethanolic extract; MLeth-2: *Mentha longifolia* L. hydro-ethanolic extract; TSeth-2: *Thymus serpyllum* L. hydro-ethanolic extract; dw: dry weight; n.d.: not detected; n.q.: not quantified. Values from the same row followed by different letters are significantly different (*p* < 0.05).

**Table 2:** Total phenolic content and antioxidant activity of *A. millefolium* L., *M. longifolia* L., and *T. serpyllum* L. extracts.

Extract	TPC (mg GAE/g dw)	RSA (%)	ABTS (mmol TE/g dw)	FRAP (μmol TE/g dw)
AMmet-1	15.62 ± 0.82 ^c^	70.77 ± 0.95 ^a^	0.14 ± 0.03 ^a^	32.20 ± 0.72 ^a^
MLmet-1	29.32 ± 1.31 ^e^	75.07 ± 0.94 ^b,c^	0.13 ± 0.04 ^a^	29.90 ± 0.76 ^a^
TSmet-1	19.09 ± 0.61 ^d^	70.51 ± 0.26 ^a^	0.06 ± 0.01 ^a^	29.87 ± 0.63 ^a^
AMeth-2	5.32 ± 0.58 ^a^	74.32 ± 0.86 ^b^	0.10 ± 0.02 ^a^	21.00 ± 0.31 ^a^
MLeth-2	17.65 ± 0.17 ^d^	76.65 ± 0.69 ^c^	0.33 ± 0.01 ^a^	10.36 ± 0.39 ^a^
TSeth-2	11.29 ± 0.37 ^b^	69.94 ± 0.90 ^a^	0.19 ± 0.01 ^a^	17.58 ± 0.49 ^a^
Ascorbic acid	n.d.	100.00 ± 0.42 ^d,^*	21.45 ± 1.53 ^b^ mmol TE/g ascorbic acid	20.07 ± 1.09 ^b^ mmol TE/g ascorbic acid

AMmet-1: *Achillea millefolium* L. hydro-methanolic extract; MLmet-1: *Mentha longifolia* L. hydro-methanolic extract; TSmet-1: *Thymus serpyllum* L. hydro-methanolic extract; AMeth-2: *Achillea millefolium* L. hydro-ethanolic extract; MLeth-2: *Mentha longifolia* L. hydro-ethanolic extract; TSeth-2: *Thymus serpyllum* L. hydro-ethanolic extract; TPC: total phenolic content; RSA: inhibition of DPPH free radical; ABTS: ABTS free radical scavenging test; FRAP: ferric-reducing antioxidant power; GAE: gallic acid equivalents; TE: trolox equivalents; dw: dry weight; n.d.: not determined; *: ascorbic acid solution 1 mg/mL. Mean values and standard deviations from the same column followed by different letters are significantly different (*p* < 0.05).

## Data Availability

Data are contained within the article.

## Supplementary Materials

The following supporting information can be downloaded at: https://www.mdpi.com/article/10.3390/molecules31091527/s1, Table S1: Summary of the results of other studies on selected phenolic compounds from the analyzed plant species; Table S2: Summary of the results of other studies on total phenolic content and antioxidant activity from the analyzed plant species. Reference [135] is cited in Supplementary Materials.
