# Supplementary material for: Evaluation of Phytochemical Constituents, Antioxidant Potential, and Toxicological Profile of Selected Medicinal Plants from Romania’s Spontaneous Flora
Source: Molecules. 2026 May 4;31(9):1527. doi: 10.3390/molecules31091527 (PMC13164594; doi:10.3390/molecules31091527)
Supplement: Supplementary file 1 [file molecules-31-01527-s001.zip › molecules-4248260-supplementary.pdf]

SUPPLEMENTARY MATERIAL

Evaluation of Phytochemical Constituents, Antioxidant Potential, and Toxicological Profile of Selected Medicinal Plants from Romania's Spontaneous Flora

Lidia-Ioana Virchea [1], Cecilia Georgescu [2,*], Adina Frum [1,*], Endre Máthé [3,4], Monica Mironescu [2], Bence Pecsenye [3], Robert Nagy [3], Oana Danci [5], Maria-Lucia Mureșan [1], Maria Totan [1] and Felicia-Gabriela Gligor [1]

[1] Faculty of Medicine, "Lucian Blaga" University of Sibiu, Lucian Blaga Str. 2A, 550169 Sibiu, Romania; lidia.virchea@ulbsibiu.ro (L.-I.V.); adina.frum@ulbsibiu.ro (A.F.); maria.muresan@ulbsibiu.ro (M.-L.M.); maria.totan@ulbsibiu.ro (M.T.); felicia.gligor@ulbsibiu.ro (F.-G.G.)

[2] Faculty of Agriculture Sciences, Food Industry and Environmental Protection, "Lucian Blaga" University of Sibiu, Dr. Ion Rațiu Str. 7-9, 550012 Sibiu, Romania; cecilia.georgescu@ulbsibiu.ro (C.G.); monica.mironescu@ulbsibiu.ro (M.M.)

[3] Institute of Nutrition Science, Faculty of Agricultural and Food Sciences and Environmental Management, University of Debrecen, Böszörményi Str. 128, 4032 Debrecen, Hungary; endre.mathe@agr.unideb.hu (E.M.); pecsenye.bence@agr.unideb.hu (B.P.); nagy.robert@agr.unideb.hu (R.N.)

[4] Department of Life Sciences, Faculty of Medicine, Vasile Goldis, Western University from Arad, L. Rebreanu Str. 86, 310414 Arad, Romania

[5] Faculty of Sciences, "Lucian Blaga" University of Sibiu, Dr. Ion Rațiu Str. 5-7, 550012 Sibiu, Romania; oana.danci@ulbsibiu.ro (O.D.)

* *Correspondence :* cecilia.georgescu@ulbsibiu.ro (C.G.); adina.frum@ulbsibiu.ro (A.F.)

Supplementary Tables

Table S1. Summary of the results of other studies on selected phenolic compounds from the analyzed plant species.

Table S2. Summary of the results of other studies on total phenolic content and antioxidant activity from the analyzed plant species.

Table S1. Summary of the results of other studies on selected phenolic compounds from the analyzed plant species.

Species	Plant part	Extraction solvent	Extraction method	Analytical techniques	Plant collection site	Phenolic compounds	Reported concentrations	Reference
A. millefolium L.	flowers	70% ethanol	Water bath extraction for 30 min, at 60°C	HPLC-MS	Cluj and Maramureș Counties, Romania	chlorogenic acid<br>rutin<br>quercetin	2317.10 µg/g plant<br>35.20 µg/g plant<br>16.00 µg/g plant	[1]
A. millefolium L.	inflorescences	70% ethanol	Water bath extraction for 30 min, at 60°C	HPLC	Sălaj County, Romania	chlorogenic acid	3069.50 µg/g dry matter	[2]
A. millefolium L.	whole plant	70% ethanol	Total extract consists of a macerate and a hydrolyzed extract	HPLC	Transylvani a, Romania	chlorogenic acid<br>caffeic acid<br>rutin<br>ferulic acid	10 µg/g dw of plant<br>460 µg/g dw of plant<br>1410 µg/g dw of plant<br>n.d.	[3]
A. millefolium L.	inflorescences	70% methanol	Ultrasonic bath extraction for 30 min, at 40°C	HPLC-PDA	Lithuania	caffeic acid	19.6 µg/g dry matter	[4]
A. millefolium L.	plant with white inflorescences	70% methanol	Ultrasonic bath extraction for 30 min, at 40°C	HPLC-PDA	Lithuania	caffeic acid<br>chlorogenic acid<br>rutin<br>quercetin	17.46 – 64.14 µg/g dw*<br>4583,70 – 28,123.17 µg/g dw*<br>176.2 – 2634.46 µg/g dw*<br>18.76 – 27.61 µg/g dw*	[5]
A. millefolium L.	herba	70% methanol	Ultrasound-assisted extraction	LC-MS	Poland	chlorogenic acid<br>caffeic acid<br>ferulic acid<br>rutin<br>quercetin	5992 µg/g sample dry matter<br>45 µg/g sample dry matter<br>460 µg/g sample dry matter<br>29 µg/g sample dry matter<br>14 µg/g sample dry matter	[6]
M. longifolia L.	aerial parts	70% ethanol	Extraction at at 60°C	LC-MS	Cluj County, Romania	rutin	8.22 µg/g dry mass	[7]

Table S1. Cont.

Species	Plant part	Extraction solvent	Extraction method	Analytical techniques	Plant collection site	Phenolic compounds	Reported concentrations	Reference
M. longifolia L.	aerial parts	70% ethanol	Soxhlet extraction	HPLC-DAD	Ifran, Morocco	caffeic acid<br>ferulic acid<br>gallic acid<br>syringic acid<br>rutin<br>quercetin	1540 µg/g dw<br>n.d.<br>1630 µg/g dw<br>30 µg/g dw<br>1300 µg/g dw<br>770 µg/g dw	[8]
M. longifolia L.	aerial parts	70% ethanol	Ultrasonic-assisted extraction	HPLC-DAD	Ifran, Morocco	caffeic acid<br>ferulic acid<br>gallic acid<br>syringic acid<br>rutin<br>quercetin	1330 µg/g dw<br>n.d.<br>n.d.<br>220 µg/g dw<br>400 µg/g dw<br>1090 µg/g dw	[8]
M. longifolia L.	aerial parts	70% ethanol	Cold maceration	HPLC-DAD	Ifran, Morocco	caffeic acid<br>ferulic acid<br>gallic acid<br>syringic acid<br>rutin<br>quercetin	n.d.<br>n.d.<br>n.d.<br>n.d.<br>7340 µg/g dw<br>3180 µg/g dw	[8]
M. longifolia L.	aerial parts	70% ethanol	Ultrasonic bath extraction for 90 min at room temperature	HPLC-DAD	Hungary	rutin<br>quercetin<br>caffeic acid<br>ferulic acid<br>gallic acid<br>syringic acid	5108 – 7571 µg/g dry herb<br>134.3 – 286.4 µg/g dry herb<br>273.3 µg/g dry herb<br>10.45 µg/g dry herb<br>n.d.<br>n.d.	[9]
T. serpyllum L.	aerial parts	60% methanol	Ultrasound extraction	LC-MS	Târgu Mureș, Romania	catechin<br>caffeic acid<br>chlorogenic acid<br>ferulic acid<br>rutin<br>quercetin	2.18 µg/g<br>109.59 µg/g<br>13.03 µg/g<br>3.64 µg/g<br>3.84 µg/g<br>6.58 µg/g	[10]
T. serpyllum L.	plant	70% ethanol	Ultrasound extraction (40°C, 20 min), re-extracted with the same solvent	HPLC-DAD	Bosnia and Herzegovina	gallic acid<br>chlorogenic acid<br>ferulic acid<br>catechin<br>rutin<br>quercetin	50 µg/g dw<br>20 µg/g dw<br>n.d.<br>50 µg/g dw<br>n.d.<br>n.d.	[11]

Table S1. Cont.

Species	Plant part	Extraction solvent	Extraction method	Analytical techniques	Plant collection site	Phenolic compounds	Reported concentrations	Refere nce
T. serpyllum L.	plant	methanol	Shaker extraction for 1 day, at 150 rpm, 40°C	HPLC	India	gallic acid<br>rutin<br>caffeic acid	1402 µg/g dw<br>875 µg/g dw<br>313 µg/g dw	[12]

LC-MS: Liquid Chromatography – Mass Spectrometry; HPLC-DAD: High Performance Liquid Chromatography – Diode Array Detector; HPLC: High Performance Liquid Chromatography; n.d.: not detected; dw: dry weight; *: depending on the plant part.

Table S2. Summary of the results of other studies on total phenolic content and antioxidant activity from the analyzed plant species.

Species	Plant part	Plant origin	Extraction solvent	TPC	Antioxidant potential	Reference
A. millefolium L.	flowering herb	Slovak Republic	80% ethanol	19.65 mg GAE/g dw	*A. millefolium* L. extract had a higher DPPH scavenging effect than *T. serpyllum* L. extract	[13]
T. serpyllum L.				119.52 mg GAE/g dw		
A. millefolium L.	aerial parts	Neamț County, Romania	70% ethanol	-	DPPH scavenging activity of 70.36% (at 0.5 dry matter/mL)	[14]
A. millefolium L.	flowers	Cluj and Maramureș Counties, Romania	70% ethanol	38.12 mg GAE/g	-	[1]
A. millefolium L.	aerial parts	Cluj County, Romania	70% ethanol	35.22 mg GAE/g dw	-	[15]
A. millefolium L.	whole plant	Transylvania, Romania	70% ethanol	134.65 mg GAE/g dw	-	[3]
A. millefolium L.	herb	purchased from a pharmacy in Bulgaria	water	2.74 – 7.92 mg GAE/g dw	FRAP activity 29.57 – 132.71 µM TE/g dw<br>ABTS free radical scavenging activity 18.59 – 125.75 µM TE/g dw	[17]
A. millefolium L.	not mentioned	Turkey	methanol	3.432 mg GAE/g	-	[18]
A. millefolium L.	aerial parts	Kosovo	80% ethanol	17.50 mg GAE/g dry matter	-	[19]
A. millefolium L.	not mentioned	Pakistan	methanol	-	DPPH radical scavenging activity 84.21% (at 300 mg/mL)	[20]
A. millefolium L.	leaves	Ooty	70% ethanol	-	DPPH inhibition by 84 - 91%, (at concentrations between 10-50 µg/mL)	[21]
A. millefolium L.	herb	Poland	70% methanol	194.59 mg GAE/g dry matter	-	[6]
A. millefolium L.	inflorescenc es, leaves, and stems	Lithuania	70% methanol	-	ABTS activity between 40.32 – 320.64 µmol TE/g dw<br>FRAP activity between 146.51 – 693.11 µmol TE/g dw	[5]
M. longifolia L.	aerial parts	Cluj County, Romania	70% ethanol	219.20 mg GAE/g dry mass	DPPH scavenging activity of 25.31%, (at 0.4 mg plant/mL extract)	[7]
M. longifolia L.	leaves	Morocco	70% ethanol	23.52 mg GAE/g plant dry weight	-	[22]

Table S2. Cont.

Species	Plant part	Plant origin	Extraction solvent	TPC	Antioxidant potential	Reference
M. longifolia L.	flowering shoots	Hungary	70% ethanol	23.39 – 67.91 mg GAE/g dry herb	-	[23]
M. longifolia L.	herb	Iran	76% methanol	-	DPPH scavenging activity of 23.88%	[24]
M. longifolia L.	aerial parts	Iran	80% methanol	5.97 to 16.06 mg GAE/g dw	-	[25]
T. serpyllum L.	aerial parts	Harghita County, Romania	50% ethanol	-	DPPH scavenging activity of about 80%	[26]
T. serpyllum L.	aerial parts	western part of Romania	70% ethanol	27.85 mg GAE/g dry material for coarse shredding vs. 45.80 mg GAE/g dry material for fine grinding	-	[27]
T. serpyllum L.	flower, leaves and stems	Lebanon	60% ethanol	13.78 mg GAE/g dw	-	[28]
T. serpyllum L.	not mentioned	India	80% ethanol	86.60 mg GAE/g dw	-	[12]
T. serpyllum L.	aerial parts	China	70% methanol	49.80 mg GAE/g dw	DPPH radical scavenging activity of 76.3%<br>FRAP value of 152.33 μmol TE/g dw<br>ABTS value of 1.41 mmol TE/g dw	[29]
T. serpyllum L.	herb	Serbia	ethanol	2.45 – 7.66 mg GAE/g of drug *	-	[30]

TPC: total phenolic content; GAE: gallic acid equivalents; dw: dry weight; DPPH: 2,2-diphenyl-1-picrylhydrazyl; -: the analysis was not included or the results were expressed in a different way and cannot be compared; *: depending on the method used for extraction, particle size of the drug, solid/solvent ratio, and time of extraction.

References

1. Benedec, D.; Popica, I.-E.; Oniga, I.; Hanganu, D.; Duma, M.; Silaghi-Dumitrescu, R.; Bischin, C.; Vlase, L. Comparative HPLC-MS Analysis of Phenolics from Achillea Distans and Achillea Millefolium and Their Bioactivity. *Studia UBB Chemia* **2015**, *LX*, 257–266.
2. Popovici, M.; Coldea, G.; Coste, A.; Pop, C.; Tămaș, M. Comparative Phytochemical Evaluation in Several Achillea Species from Romania. *Farmacia* **2021**, *69*, 928–933, doi:10.31925/farmacia.2021.5.15.
3. Bobis, O.; Dezmirean, D.S.; Tomos, L.; Chirila, F.; Marghitas, L.Al. Influence of Phytochemical Profile on Antibacterial Activity of Different Medicinal Plants against Gram-Positive and Gram-Negative Bacteria. *Appl Biochem Microbiol* **2015**, *51*, 113–118, doi:10.1134/S0003683815010044.
4. Radušienė, J.; Karpavičienė, B.; Raudone, L.; Vilkickyte, G.; Çırak, C.; Seyis, F.; Yayla, F.; Marksa, M.; Rimkienė, L.; Ivanauskas, L. Trends in Phenolic Profiles of Achillea Millefolium from Different Geographical Gradients. *Plants* **2023**, *12*, 746, doi:10.3390/plants12040746.
5. Raudone, L.; Vilkickyte, G.; Marksa, M.; Radusiene, J. Comparative Phytoprofiling of Achillea Millefolium Morphotypes: Assessing Antioxidant Activity, Phenolic and Triterpenic Compounds Variation across Different Plant Parts. *Plants* **2024**, *13*, 1043, doi:10.3390/plants13071043.
6. Konarska, A.; Weryszko-Chmielewska, E.; Materska, M.; Sulborska-Różycka, A.; Dmitruk, M.; Chilczuk, B. Phenolic Compounds in Flowers and Herb of Achillea Millefolium L.: Histochemical and Phytochemical Studies. *Molecules* **2025**, *30*, 2084, doi:10.3390/molecules30092084.
7. Benedec, D.; Vlase, L.; Oniga, I.; Mot, A.C.; Silaghi-Dumitrescu, R.; Hanganu, D.; Tiperciuc, B.; Crişan, G. LC-MS Analysis and Antioxidantactivity of Phenolic Compounds from Two Indigenous Species of Mentha. Note I. *Farmacia* **2013**, *61*, 262–267.
8. Tourabi, M.; Faiz, K.; Ezzouggari, R.; Louasté, B.; Merzouki, M.; Dauelbait, M.; Bourhia, M.; Almaary, K.S.; Siddique, F.; Lyoussi, B.; et al. Optimization of Extraction Process and Solvent Polarities to Enhance the Recovery of Phytochemical Compounds, Nutritional Content, and Biofunctional Properties of Mentha Longifolia L. Extracts. *Bioresour. Bioprocess.* **2025**, *12*, 24, doi:10.1186/s40643-025-00859-8.
9. Patonay, K.; Korózs, M.; Murányi, Z.; Pénzesné Kónya, E. Polyphenols in Northern Hungarian Mentha Longifolia (L.) L. Treated with Ultrasonic Extraction for Potential Oenological Uses. *Turk J Agric For* **2017**, *41*, 208–217, doi:10.3906/tar-1701-61.

10. Varga, E.; Bardocz, A.; Belák, Á.; Maráz, A.; Boros, B.; Felinger, A.; Böszörményi, A.; Horváth, G. Antimicrobial Activity and Chemical Ccomposition of Thyme Essential Oils and the Polyphenolic Content of Different Thymus Extracts. *Farmacia* **2015**, *63*, 357–361.
11. Ibragic, S.; Djukic, A.; Murga, M.; Tuka, P.; Radoncic, I.; Bozur, I.; Dizdar, M.; Culum, D.; Topcagic, A. Phenolic Composition, Cholinesterase Inhibition, and in Silico Study of Traditional Medicinal Plants from Bosnia And Herzegovina. *Natural Product Research* **2025**, 1–10, doi:10.1080/14786419.2025.2542309.
12. Shahar, B.; Indira, A.; Santosh, O.; Dolma, N.; Chongtham, N. Nutritional Composition, Antioxidant Activity and Characterization of Bioactive Compounds from Thymus Serpyllum L.: An Underexploited Wild Aromatic Plant. *Measurement: Food* **2023**, *10*, 100092, doi:10.1016/j.meafoo.2023.100092.
13. Ivanišová, E.; Horňák, M.; Čech, M.; Harangozo, Ľ.; Kačániová, M.; Grygorieva, O.; Kowalczewski, P.Ł. Polyphenol Content, Mineral Compounds Composition, Antimicrobial and Antioxidant Activities of Selected Medicinal Herbs from Slovak Republic. *Applied Sciences* **2023**, *13*, 1918, doi:10.3390/app13031918.
14. Alexandru, V.; Balan, M.; Gaspar, A.; Craciunescu, O.; Moldovan, L. Studies on the Antioxidant Activity, Phenol and Flavonoid Contents of Some Selected Romanian Medicinal Plants Used for Wound Healing. *Romanian Biotechnological Letters* **2007**, *12*, 3467–3472.
15. Gavan, A.; Colobatiu, L.; Hanganu, D.; Bogdan, C.; Olah, N.K.; Achim, M.; Mirel, S. Development and Evaluation of Hydrogel Wound Dressings Loaded with Herbal Extracts. *Processes* **2022**, *10*, 242, doi:10.3390/pr10020242.
16. Alexandru, V.; Gaspar, A.; Savin, S.; Toma, A.; Tatia, R.; Gille, E. Phenolic Content, Antioxidant Activity and Effect on Collagen Synthesis of a Traditional Wound Healing Polyherbal Formula. *Studia Universitatis "Vasile Goldiş", Seria Ştiinţele Vieţii* **2015**, *25*, 41–46.
17. Georgieva, L.; Gadjalova, A.; Mihaylova, D.; Pavlov, A. Achillea Millefolium L. – Phytochemical Profile and in Vitro Antioxidant Activity. *International Food Research Journal* **2015**, *22*, 1347–1352.
18. Ayhan, N.K.; Tunc, M.G.K.; Noma, S.A.A.; Kurucay, A.; Ates, B. Characterization of the Antioxidant Activity, Total Phenolic Content, Enzyme Inhibition, and Anticancer Properties of *Achillea Millefolium* L. (Yarrow). *Instrumentation Science & Technology* **2022**, *50*, 654–667, doi:10.1080/10739149.2022.2073369.
19. Faiku, F.; Haziri, A.; Faiku, F.; Faiku, B. Composition of Some Macro and Micro Elements, Polyphenol Content, Antimicrobial and Antioxidant Activity of Achillea Millefolium (L.) Grown in Kosovo. *chemija* **2025**, *36*, 69–77, doi:10.6001/chemija.2025.36.2.3.
20. Adil, M.; Dastagir, G.; Quddoos, A.; Naseer, M.; Filimban, F.Z. HPLC Analysis, Genotoxic and Antioxidant Potential of Achillea Millefolium L. and

Chaerophyllum Villosum Wall Ex. Dc. *BMC Complement Med Ther* **2024**, *24*, 91, doi:10.1186/s12906-024-04344-1.
21. Devadharshini, R.; Kavya, S.V.; Gowrishankar, M.; Renuka, K.; Muthukumaran, P. In Vitro Evaluation of Total Phenol and Antioxidant Activity of Achillea Millefolium and Stevia Rebaudiana. *Journal of Chemical and Pharmaceutical Research* **2021**, *13*, 01–08.
22. Tourabi, M.; Metouekel, A.; Ghouizi, A.E.L.; Jeddi, M.; Nouioura, G.; Laaroussi, H.; Hosen, Md.E.; Benbrahim, K.F.; Bourhia, M.; Salamatullah, A.M.; et al. Efficacy of Various Extracting Solvents on Phytochemical Composition, and Biological Properties of Mentha Longifolia L. Leaf Extracts. *Sci Rep* **2023**, *13*, 18028, doi:10.1038/s41598-023-45030-5.
23. Patonay, K.; Szalontai, H.; Csugány, J.; Szabó-Hudák, O.; Kónya, E.P.; Németh, É.Z. Comparison of Extraction Methods for the Assessment of Total Polyphenol Content and in Vitro Antioxidant Capacity of Horsemint (Mentha Longifolia (L.) L.). *Journal of Applied Research on Medicinal and Aromatic Plants* **2019**, *15*, 100220, doi:10.1016/j.jarmap.2019.100220.
24. Fahmideh, L.; Mazaraie, A.; Tavakoli, M. Total Phenol/Flavonoid Content, Antibacterial and DPPH Free Radical Scavenging Activities of Medicinal Plants. *Journal of Agricultural Science and Technology* **2019**, *21*, 1459–1471.
25. Tabar, F.M.; Fathi, S.; Shameh, S.; Alirezalu, A. Phytochemicals and Antioxidant Activity of Mentha Longifolia Ecotypes. *Russ J Plant Physiol* **2025**, *72*, 80, doi:10.1134/S1021443724610553.
26. Papp, N.; Sali, N.; Csepregi, R.; Tóth, M.; Gyergyák, K.; Dénes, T.; Bartha, S.G.; Varga, E.; Kaszás, A.; Kőszegi, T. Antioxidant Potential of Some Plants Used in Folk Medicine in Romania. *Farmacia* **2019**, *67*, 323–330, doi:10.31925/farmacia.2019.2.18.
27. Horablaga, N.M.; Cozma, A.; Alexa, E.; Obistioiu, D.; Cocan, I.; Poiana, M.-A.; Lalescu, D.; Pop, G.; Imbrea, I.M.; Buzna, C. Influence of Sample Preparation/Extraction Method on the Phytochemical Profile and Antimicrobial Activities of 12 Commonly Consumed Medicinal Plants in Romania. *Applied Sciences* **2023**, *13*, 2530, doi:10.3390/app13042530.
28. Predescu, C.; Papuc, C.; Ștefan, G.; Petcu, C. Phenolics Content, Antioxidant and Antimicrobial Activities of Some Extracts Obtained from Romanian Summer Savory and Lebanon Wild Thyme. *Scientific Works. Series C. Veterinary Medicine* **2020**, *LXVI*, 17–22.
29. Yang, R.; Dong, Y.; Gao, F.; Li, J.; Stevanovic, Z.D.; Li, H.; Shi, L. Comprehensive Analysis of Secondary Metabolites of Four Medicinal Thyme Species Used in Folk Medicine and Their Antioxidant Activities In Vitro. *Molecules* **2023**, *28*, 2582, doi:10.3390/molecules28062582.
30. Jovanović, A.; Skrt, M.; Petrović, P.; Častvan, I.; Zdunić, G.; Šavikin, K.; Bugarski, B. Ethanol Thymus Serpyllum Extracts: Evaluation of Extraction Conditions via Total

Polyphenol Content and Radical Scavenging Activity. *Lekovite Sirovine* **2019**, *39*, doi:10.5937/leksir1939023j.
